# Crystal structure and Hirshfeld surface analysis of (*E*)-1-(2,4-di­methyl­furan-3-yl)-3-phenyl­prop-2-en-1-one

**DOI:** 10.1107/S2056989023006084

**Published:** 2023-07-14

**Authors:** Ali N. Khalilov, Victor N. Khrustalev, Aida I. Samigullina, Mehmet Akkurt, Rovnag M. Rzayev, Ajaya Bhattarai, İbrahim G. Mamedov

**Affiliations:** a "Composite Materials" Scientific Research Center, Azerbaijan State Economic University (UNEC), H. Aliyev str. 135, Az 1063, Baku, Azerbaijan; b Department of Chemistry, Baku State University, Z. Khalilov str. 23, Az, 1148, Baku, Azerbaijan; c Peoples’ Friendship University of Russia (RUDN University), Miklukho-Maklay St.6, Moscow, 117198, Russian Federation; d N. D. Zelinsky Institute of Organic Chemistry RAS, Leninsky Prosp. 47, Moscow, 119991, Russian Federation; e Department of Physics, Faculty of Sciences, Erciyes University, 38039 Kayseri, Türkiye; f Department of Chemistry, M.M.A.M.C (Tribhuvan University) Biratnagar, Nepal; Katholieke Universiteit Leuven, Belgium

**Keywords:** crystal structure, 2,4-di­methyl­furan, chalcones, hydrogen bond, C—H⋯π inter­actions, Hirshfeld surface analysis

## Abstract

The title compound, C_15_H_14_O_2_, adopts an *E* configuration about the C=C double bond. The furan ring is inclined to the phenyl ring by 12.03 (9)°. In the crystal, pairs of mol­ecules are linked by C—H⋯O hydrogen bonds, forming dimers with *R* ^2^_2_(14) ring motifs. The mol­ecules are connected *via* C—H⋯π inter­actions, forming a three dimensional network. No π–π inter­actions are observed.

## Abstract 2

In the crystal, pairs of mol­ecules are linked by C—H⋯O hydrogen bonds, forming dimers with 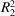 (14) ring motifs. Mol­ecules are connected *via* C—H⋯π inter­actions forming a three-dimensional network.

## 1. Chemical context

Various C—C, C—N, C—S and C—O bond-formation reactions are keystones in organic synthesis. The application of such reactions has been expanded considerably, extending these approaches in different branches of chemistry, including green, medicinal, pharmaceutical and natural products chemistry, material science, supra­molecular chemistry (Asadov *et al.*, 2003 ▸; Çelik *et al.*, 2023 ▸; Chalkha *et al.*, 2023 ▸; Gurbanov *et al.*, 2020 ▸; Zubkov *et al.*, 2018 ▸). α,β-Unsaturated ketones containing ar­yl–aryl or ar­yl–alkyl groups at both ends are known as chalcones or enones. There have been several important examples of enone derivatives used as target products and also as synthetic inter­mediates. Many natural compounds containing enone moieties, such as cyanthiwigin U, (+)-cepharamine, phorbol and grandisine G, have been the object of a total synthesis (Cuthbertson & Taylor, 2013 ▸; Kawamura *et al.*, 2016 ▸). These compounds have been obtained by many solvent-assisted or solvent-free methods. The enone moiety is a widespread structural motif of various synthetic biologically active compounds, possessing enzyme inhibitory, anti­cancer and anti­microbial activity (Poustforoosh *et al.*, 2022 ▸; Tapera *et al.*, 2022 ▸; Sarkı *et al.*, 2023 ▸).

In a continuation of our investigations in heterocyclic systems exhibiting biological activity and in the framework of ongoing structural studies (Maharramov *et al.*, 2021 ▸, 2022 ▸), we report herein the crystal structure and Hirshfeld surface analysis of the title compound, (*E*)-1-(2,4-di­methyl­furan-3-yl)-3-phenyl­prop-2-en-1-one. 
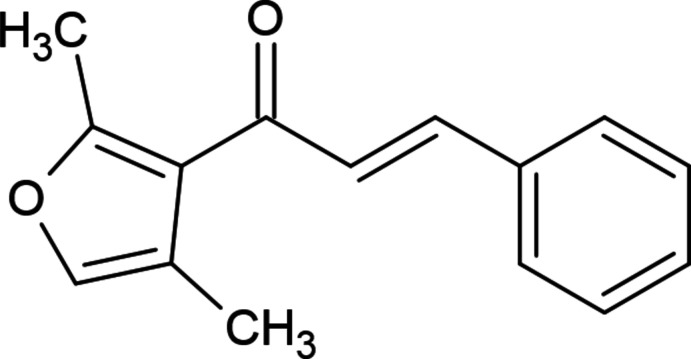


## 2. Structural commentary

**Figure 1:**
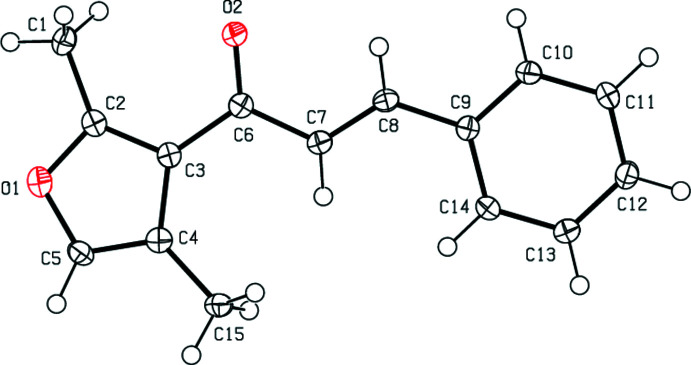
The mol­ecular structure of the title compound, showing the atom labelling and displacement ellipsoids drawn at the 50% probability level.

As seen as Fig. 1 ▸, the title compound adopts an *E* configuration about the C=C double bond. The whole mol­ecule is nearly planar. The furan ring (O1/C2–C5) is inclined to the phenyl ring (C9–C14) by 12.03 (9)°. The torsion angles are C2—C3—C6—O2 = 14.5 (2), C2—C3—C6—C7 = −164.79 (15), C3—C6—C7—C8 = −173.80 (15), C6—C7—C8—C9 = 179.30 (15) and C7—C8—C9—C10 = 172.52 (16)°. The geometrical parameter values of the the title compound are in agreement with those reported for similar compounds in the *Database survey* section.

## 3. Supra­molecular features and Hirshfeld surface analysis

**Figure 2:**
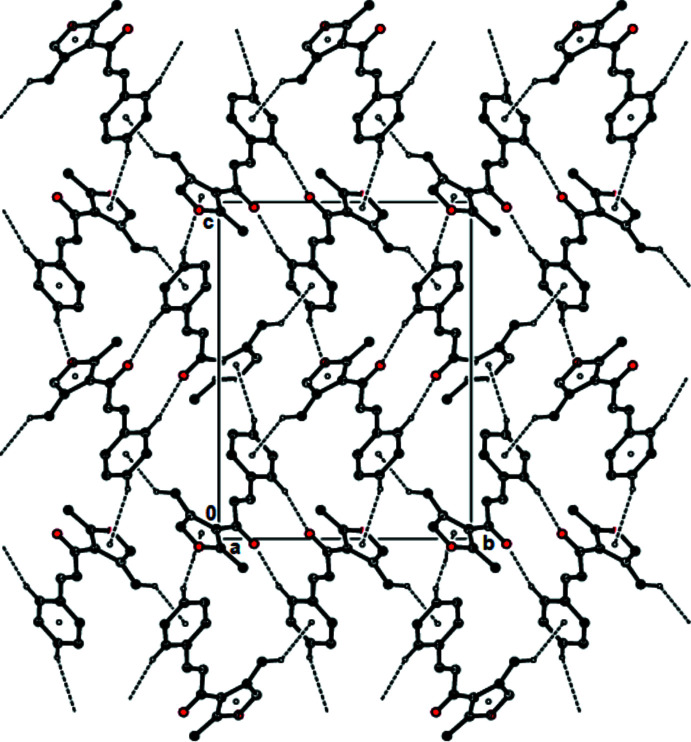
View of the C—H⋯O hydrogen bonds and C—H⋯π inter­actions of the title compound down the *a* axis. Only the H atoms involved in these inter­actions have been included.

**Figure 4:**
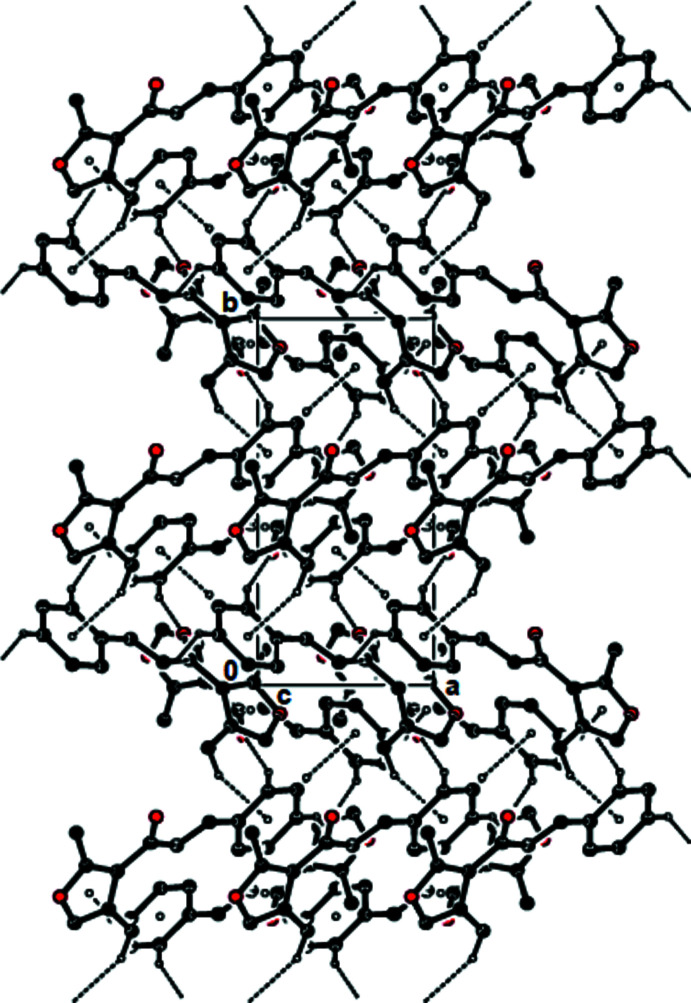
View of the C—H⋯O hydrogen bonds and C—H⋯π inter­actions of the title compound down the *c* axis. Only the H atoms involved in these inter­actions have been included.

In the crystal, pairs of mol­ecules are linked by C—H⋯O hydrogen bonds, forming dimers with 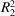 (14) ring motifs (Bernstein *et al.*, 1995 ▸; Table 1 ▸; Figs. 2 ▸ and 3 ▸). The mol­ecules are connected *via* C—H⋯π inter­actions, forming a three-dimensional network (Table 1 ▸; Fig. 4 ▸). No π–π inter­actions are observed.

**Figure 5:**
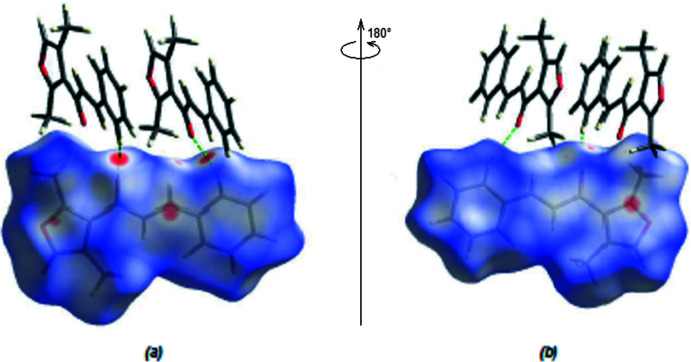
(*a*) Front and (*b*) back sides of the three-dimensional Hirshfeld surface of the title compound mapped over *d*_norm_, with a fixed colour scale of −0.1518 to +1.1567 a.u.

*CrystalExplorer17.5* (Spackman *et al.*, 2021 ▸) was used to compute Hirshfeld surfaces of the title mol­ecule and two-dimensional fingerprints. The *d*_norm_ mappings for the title compound were performed in the range −0.1518 (red) to +1.1567 (blue) a.u. On the *d*_norm_ surfaces, bright-red spots indicate the locations of the C—H⋯O inter­actions and O⋯C/C⋯O contacts (Tables 1 ▸ and 2 ▸; Fig. 5 ▸ *a,b*).

**Figure 6:**
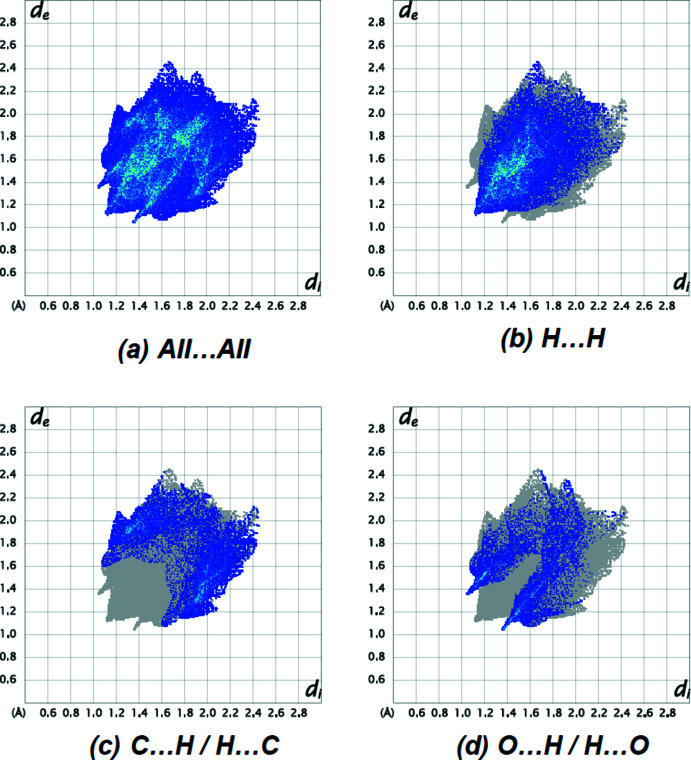
The two-dimensional fingerprint plots of the title compound, showing (*a*) all inter­actions, and delineated into (*b*) H⋯H, (*c*) C⋯H/H⋯C and (*d*) O⋯H/H⋯O inter­actions. [*d*_e_ and *d*_i_ represent the distances from a point on the Hirshfeld surface to the nearest atoms outside (external) and inside (inter­nal) the surface, respectively].

The most important inter­atomic contact is H⋯H (51.1%; Fig. 6 ▸ *b*) as it makes the highest contribution to the crystal packing. The C⋯H/H⋯C (Fig. 6 ▸ *c*; 25.3%), O⋯H/H⋯O (Fig. 6 ▸ *d*; 15.9%), C⋯C (5.1%) and O⋯C/C⋯O (2.5%) contacts have little directional influence on the mol­ecular packing.

## 4. Database survey

A search of the Cambridge Structural Database (CSD, Version 5.43, last update November 2022; Groom *et al.*, 2016 ▸) for the ‘1-(furan-3-yl)-3-phenyl­prop-2-en-1-one’ skeleton of the title compound yielded one hit, 1-(3-fur­yl)-3-[3-(tri­fluoro­meth­yl)phen­yl]prop-2-en-1-one (CSD refcode KUDNAA; Bąkowicz *et al.*, 2015 ▸). When the positions of the furan and phenyl rings are switched, 1-(3-chloro­phen­yl)-3-(3-fur­yl)prop-2-en-1-one (NUQFOW; Zingales *et al.* 2015 ▸), (*E*)-3-(2-fur­yl)-1-phenyl­prop-2-en-1-one (NOTCUW01; Vázquez-Vuelvas *et al.* 2015 ▸) are the most similar structures.

In KUDNAA, mol­ecules are linked by inter­molecular C—H⋯O inter­actions, forming zigzag chains with *C*(5) motifs along the *b*-axis direction. In addition, mol­ecules are connected by face-to-face π–π stacking inter­actions [centroid–centroid distances = 3.926 (3) and 3.925 (2) Å] between the opposing benzene and furan rings of the mol­ecules along the *c*-axis direction. In NUQFOW, the mol­ecule exhibits a non-planar geometry, the furan ring being inclined to the benzene ring by 50.52 (16)°. In the crystal of NUQFOW, mol­ecules stack along the *a*-axis; however, there are no significant inter­molecular inter­actions present. In NOTCUW01, the mol­ecule also adopts an *E* configuration about the C=C double bond and the furan and phenyl rings are inclined to one another by 24.07 (7)°. In the crystal of NOTCUW01, mol­ecules are connected by weak C—H⋯O hydrogen bonds and C—H⋯π inter­actions, forming ribbons extending along the *c*-axis direction.

## 5. Synthesis and crystallization

To a solution of 1-(2,4-di­methyl­furan-3-yl)ethan-1-one (2 g, 14.5 mmol) in ethanol (10 mL), were added 10 mL of aqueous solution of sodium hydroxide (0.65 g, 16.3 mmol) and the mixture was stirred at room temperature for 2 h. Then benzaldehyde (1.73 g, 16.3 mmol) was added to the vigorously stirred reaction mixture and it was left overnight. The precipitated crystals were separated by filtration and recrystallized from an ethanol/water (1:1) solution (yield 90%; m.p. 349–350 K).

^1^H NMR (300 MHz, DMSO-*d*_6_, ppm): 2.1 (*s*, 3H, CH_3_); 2.5 (*s*, 3H, CH_3_); 7.2 (*d*, 1H, =CH, ^3^ *J*_H–H_ = 15.8 Hz); 7.3 (*s*, 1H, fur.), 7.4 (*m*, 3H, arom.), 7.5 (*d*, 1H, =CH, ^3^ *J*_H–H_ = 15.8 Hz); 7.8 (*m*, 2H, arom.). ^13^C NMR (75 MHz, DMSO-*d*_6_, ppm): 10.3 (CH_3_), 15.0 (CH_3_), 120.4 (C_quat._), 122.9 (C_quat._), 126.3 (=CH), 128.9 (CH, arom.), 129.4 (CH, arom.), 130.9 (CH, arom.), 134.8 (C_quat._), 139.0 (CH, furan), 142.8 (=CH), 158.2 (C_quat._), 187.7 (CO).

## 6. Refinement

Crystal data, data collection and structure refinement details are summarized in Table 3 ▸. All C-bound H atoms were placed at calculated positions and refined using a riding model, with C—H = 0.95 and 0.98 Å, and with *U*_iso_(H) = 1.2 or 1.5*U*_eq_(C).

## Supplementary Material

Crystal structure: contains datablock(s) I. DOI: 10.1107/S2056989023006084/vm2287sup1.cif

Structure factors: contains datablock(s) I. DOI: 10.1107/S2056989023006084/vm2287Isup2.hkl

Supporting information file. DOI: 10.1107/S2056989023006084/vm2287Isup3.cml

CCDC reference: 2280559

Additional supporting information:  crystallographic information; 3D view; checkCIF report

## Figures and Tables

**Figure 3:**
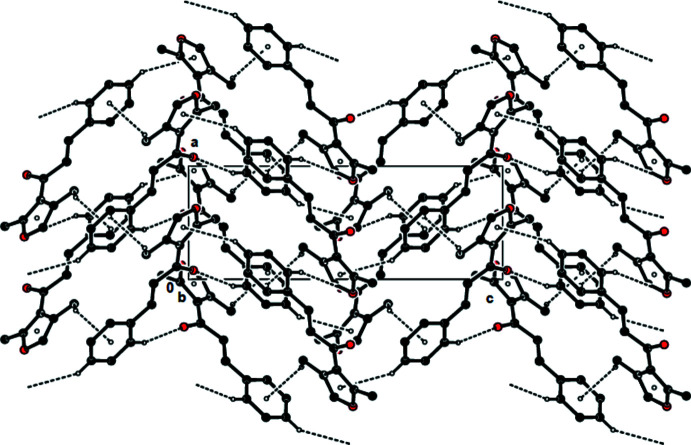
View of the C—H⋯O hydrogen bonds and C—H⋯π inter­actions of the title compound down the *b* axis. Only the H atoms involved in these inter­actions have been included.

**Table 1:** Hydrogen-bond geometry (Å, °) *Cg*1 and *Cg*2 are the centroids of the furan (O1/C2–C5) and phenyl (C9–C14) rings, respectively.

*D*—H⋯*A*	*D*—H	H⋯*A*	*D*⋯*A*	*D*—H⋯*A*
C10—H10⋯O2^i^	0.95	2.52	3.413 (2)	157
C12—H12⋯*Cg*1^ii^	0.95	2.91	3.7339 (19)	146
C15—H15*B*⋯*Cg*2^iii^	0.98	2.85	3.6366 (19)	137

Symmetry codes: (i) 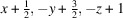 ; (ii) 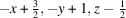 ; (iii) 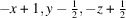 .

**Table 2:** Summary of short inter­atomic contacts (Å) in the title compound

H1*B*⋯H8	2.57	−1 + *x*, *y*, *z*
H1*C*⋯H13	2.52	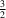 − *x*, 1 − *y*, 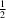 + *z*
H1*B*⋯H8	2.47	− 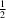 + *x*, 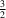 − *y*, 1 − *z*
H1*A*⋯H10	2.39	− 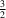 + *x*, 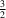 − *y*, 1 − *z*
C12⋯H5	2.94	1 − *x*, 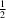 + *y*, 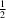 − *z*
H13⋯H1*A*	2.49	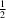 − *x*, 1 − *y*, − 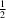 + *z*
C15⋯H11	3.05	2 − *x*, − 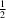 + *y*, 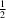 − *z*

**Table 3:** Experimental details

Crystal data	
Chemical formula	C_15_H_14_O_2_
*M*_r_	226.26
Crystal system, space group	Orthorhombic, *P*2_1_2_1_2_1_
Temperature (K)	100
*a*, *b*, *c* (Å)	5.84787 (5), 12.18109 (9), 16.24568 (15)
*V* (Å^3^)	1157.24 (2)
*Z*	4
Radiation type	Cu *K*α
μ (mm^−1^)	0.68
Crystal size (mm)	0.24 × 0.20 × 0.18

Data collection	
Diffractometer	XtaLAB Synergy, Dualflex, HyPix
Absorption correction	Multi-scan (*CrysAlis PRO*; Rigaku OD, 2021 ▸)
*T*_min_, *T*_max_	0.579, 1.000
No. of measured, independent and observed [*I* > 2σ(*I*)] reflections	12000, 2410, 2382
*R*_int_	0.031
(sin θ/λ)_max_ (Å^−1^)	0.634

Refinement	
*R*[*F* ^2^ > 2σ(*F* ^2^)], *wR*(*F* ^2^), *S*	0.030, 0.081, 1.06
No. of reflections	2410
No. of parameters	157
H-atom treatment	H-atom parameters constrained
Δρ_max_, Δρ_min_ (e Å^−3^)	0.18, −0.24
Absolute structure	Flack *x* determined using 940 quotients [(*I* ^+^)−(*I* ^−^)]/[(*I* ^+^)+(*I* ^−^)] (Parsons *et al.*, 2013 ▸)
Absolute structure parameter	0.16 (7)

Computer programs: *CrysAlis PRO* (Rigaku OD, 2021 ▸), *SHELXT2014/5* (Sheldrick, 2015*a* ▸), *SHELXL2018/3* (Sheldrick, 2015*b* ▸), *ORTEP-3 for Windows* (Farrugia, 2012 ▸) and *PLATON* (Spek, 2020 ▸).

## supplementary crystallographic information

### Crystal data

**Table:**

C_15_H_14_O_2_	*D*_x_ = 1.299 Mg m^−^^3^
*M**_r_* = 226.26	Cu *K*α radiation, λ = 1.54184 Å
Orthorhombic, *P*2_1_2_1_2_1_	Cell parameters from 10780 reflections
*a* = 5.84787 (5) Å	θ = 4.5–77.7°
*b* = 12.18109 (9) Å	µ = 0.68 mm^−^^1^
*c* = 16.24568 (15) Å	*T* = 100 K
*V* = 1157.24 (2) Å^3^	Prism, colourless
*Z* = 4	0.24 × 0.20 × 0.18 mm
*F*(000) = 480	

### Data collection

**Table:**

XtaLAB Synergy, Dualflex, HyPix diffractometer	2382 reflections with *I* > 2σ(*I*)
Radiation source: micro-focus sealed X-ray tube	*R*_int_ = 0.031
φ and ω scans	θ_max_ = 77.8°, θ_min_ = 4.5°
Absorption correction: multi-scan (CrysAlisPro; Rigaku OD, 2021)	*h* = −7→6
*T*_min_ = 0.579, *T*_max_ = 1.000	*k* = −15→15
12000 measured reflections	*l* = −20→20
2410 independent reflections	

### Refinement

**Table:**

Refinement on *F*^2^	Hydrogen site location: inferred from neighbouring sites
Least-squares matrix: full	H-atom parameters constrained
*R*[*F*^2^ > 2σ(*F*^2^)] = 0.030	*w* = 1/[σ^2^(*F*_o_^2^) + (0.0426*P*)^2^ + 0.3199*P*] where *P* = (*F*_o_^2^ + 2*F*_c_^2^)/3
*wR*(*F*^2^) = 0.081	(Δ/σ)_max_ < 0.001
*S* = 1.06	Δρ_max_ = 0.18 e Å^−^^3^
2410 reflections	Δρ_min_ = −0.24 e Å^−^^3^
157 parameters	Extinction correction: SHELXL, Fc^*^=kFc[1+0.001xFc^2^λ^3^/sin(2θ)]^-1/4^
0 restraints	Extinction coefficient: 0.0046 (4)
Primary atom site location: difference Fourier map	Absolute structure: Flack *x* determined using 940 quotients [(*I*^+^)-(*I*^-^)]/[(*I*^+^)+(*I*^-^)] (Parsons *et al.*, 2013)
Secondary atom site location: difference Fourier map	Absolute structure parameter: 0.16 (7)

### Special details

**Table:**

Experimental. CrysAlisPro 1.171.41.117a (Rigaku OD, 2021) Empirical absorption correction using spherical harmonics, implemented in SCALE3 ABSPACK scaling algorithm.
Geometry. All esds (except the esd in the dihedral angle between two l.s. planes) are estimated using the full covariance matrix. The cell esds are taken into account individually in the estimation of esds in distances, angles and torsion angles; correlations between esds in cell parameters are only used when they are defined by crystal symmetry. An approximate (isotropic) treatment of cell esds is used for estimating esds involving l.s. planes.

### Fractional atomic coordinates and isotropic or equivalent isotropic displacement parameters (Å^2^)

**Table:**

	*x*	*y*	*z*	*U*_iso_*/*U*_eq_	
O1	−0.1250 (2)	0.42159 (10)	0.52334 (8)	0.0225 (3)	
O2	0.4218 (2)	0.63977 (10)	0.51486 (7)	0.0208 (3)	
C1	−0.0327 (3)	0.59606 (14)	0.58548 (11)	0.0211 (4)	
H1A	−0.1936	0.5903	0.6018	0.032*	
H1B	−0.0064	0.6672	0.5590	0.032*	
H1C	0.0647	0.5898	0.6343	0.032*	
C2	0.0234 (3)	0.50662 (13)	0.52698 (10)	0.0174 (3)	
C3	0.1991 (3)	0.49066 (13)	0.47138 (10)	0.0162 (3)	
C4	0.1518 (3)	0.38801 (13)	0.43028 (10)	0.0183 (4)	
C5	−0.0431 (3)	0.34981 (13)	0.46379 (11)	0.0197 (3)	
H5	−0.1150	0.2828	0.4488	0.024*	
C6	0.3897 (3)	0.56901 (13)	0.46209 (10)	0.0165 (3)	
C7	0.5382 (3)	0.56193 (14)	0.38864 (10)	0.0184 (3)	
H7	0.5012	0.5122	0.3456	0.022*	
C8	0.7247 (3)	0.62533 (13)	0.38247 (10)	0.0169 (3)	
H8	0.7528	0.6742	0.4269	0.020*	
C9	0.8896 (3)	0.62758 (13)	0.31489 (10)	0.0162 (3)	
C10	1.0585 (3)	0.70888 (13)	0.31532 (11)	0.0193 (3)	
H10	1.0611	0.7615	0.3584	0.023*	
C11	1.2225 (3)	0.71374 (14)	0.25365 (11)	0.0217 (4)	
H11	1.3360	0.7695	0.2547	0.026*	
C12	1.2207 (3)	0.63690 (14)	0.19019 (11)	0.0206 (4)	
H12	1.3331	0.6400	0.1480	0.025*	
C13	1.0538 (3)	0.55571 (14)	0.18888 (11)	0.0214 (4)	
H13	1.0518	0.5033	0.1456	0.026*	
C14	0.8901 (3)	0.55079 (14)	0.25044 (11)	0.0207 (3)	
H14	0.7771	0.4948	0.2490	0.025*	
C15	0.2820 (3)	0.32838 (14)	0.36480 (11)	0.0222 (4)	
H15A	0.2637	0.3667	0.3122	0.033*	
H15B	0.2231	0.2534	0.3598	0.033*	
H15C	0.4444	0.3260	0.3796	0.033*	

### Atomic displacement parameters (Å^2^)

**Table:**

	*U*^11^	*U*^22^	*U*^33^	*U*^12^	*U*^13^	*U*^23^
O1	0.0189 (6)	0.0244 (6)	0.0242 (6)	−0.0017 (5)	0.0021 (5)	0.0025 (5)
O2	0.0195 (6)	0.0223 (6)	0.0207 (6)	−0.0032 (5)	0.0029 (5)	−0.0048 (5)
C1	0.0186 (7)	0.0235 (8)	0.0211 (8)	0.0027 (7)	0.0039 (7)	0.0001 (6)
C2	0.0161 (7)	0.0175 (7)	0.0187 (8)	0.0009 (6)	−0.0019 (6)	0.0034 (6)
C3	0.0155 (7)	0.0171 (7)	0.0161 (7)	0.0005 (6)	−0.0013 (6)	0.0017 (6)
C4	0.0190 (8)	0.0175 (8)	0.0184 (7)	0.0015 (6)	−0.0016 (6)	0.0009 (6)
C5	0.0190 (7)	0.0160 (7)	0.0241 (8)	−0.0030 (6)	−0.0008 (7)	−0.0005 (6)
C6	0.0155 (7)	0.0167 (7)	0.0172 (7)	0.0020 (6)	−0.0014 (6)	0.0011 (6)
C7	0.0189 (7)	0.0185 (7)	0.0177 (7)	−0.0006 (6)	0.0009 (6)	−0.0025 (6)
C8	0.0181 (7)	0.0154 (7)	0.0172 (7)	0.0021 (6)	−0.0007 (6)	−0.0011 (6)
C9	0.0155 (7)	0.0170 (7)	0.0160 (7)	0.0009 (6)	0.0002 (6)	0.0010 (6)
C10	0.0192 (8)	0.0182 (7)	0.0204 (8)	−0.0017 (7)	0.0003 (7)	−0.0035 (6)
C11	0.0192 (8)	0.0208 (8)	0.0250 (8)	−0.0056 (7)	0.0037 (7)	−0.0011 (7)
C12	0.0193 (8)	0.0224 (8)	0.0200 (8)	0.0008 (7)	0.0044 (7)	0.0006 (7)
C13	0.0225 (8)	0.0218 (8)	0.0199 (8)	−0.0018 (7)	0.0024 (7)	−0.0047 (7)
C14	0.0193 (7)	0.0201 (8)	0.0226 (8)	−0.0055 (7)	0.0023 (7)	−0.0042 (6)
C15	0.0222 (8)	0.0187 (8)	0.0258 (9)	−0.0009 (7)	0.0014 (7)	−0.0060 (7)

### Geometric parameters (Å, º)

**Table:**

O1—C2	1.352 (2)	C8—C9	1.461 (2)
O1—C5	1.389 (2)	C8—H8	0.9500
O2—C6	1.230 (2)	C9—C10	1.399 (2)
C1—C2	1.482 (2)	C9—C14	1.404 (2)
C1—H1A	0.9800	C10—C11	1.388 (2)
C1—H1B	0.9800	C10—H10	0.9500
C1—H1C	0.9800	C11—C12	1.392 (2)
C2—C3	1.382 (2)	C11—H11	0.9500
C3—C4	1.444 (2)	C12—C13	1.390 (2)
C3—C6	1.475 (2)	C12—H12	0.9500
C4—C5	1.346 (2)	C13—C14	1.385 (2)
C4—C15	1.496 (2)	C13—H13	0.9500
C5—H5	0.9500	C14—H14	0.9500
C6—C7	1.478 (2)	C15—H15A	0.9800
C7—C8	1.340 (2)	C15—H15B	0.9800
C7—H7	0.9500	C15—H15C	0.9800
			
C2—O1—C5	106.96 (13)	C7—C8—H8	116.4
C2—C1—H1A	109.5	C9—C8—H8	116.4
C2—C1—H1B	109.5	C10—C9—C14	118.27 (15)
H1A—C1—H1B	109.5	C10—C9—C8	118.39 (14)
C2—C1—H1C	109.5	C14—C9—C8	123.32 (15)
H1A—C1—H1C	109.5	C11—C10—C9	120.97 (15)
H1B—C1—H1C	109.5	C11—C10—H10	119.5
O1—C2—C3	109.92 (14)	C9—C10—H10	119.5
O1—C2—C1	116.68 (14)	C10—C11—C12	120.04 (16)
C3—C2—C1	133.39 (16)	C10—C11—H11	120.0
C2—C3—C4	106.33 (14)	C12—C11—H11	120.0
C2—C3—C6	122.53 (15)	C13—C12—C11	119.66 (16)
C4—C3—C6	131.14 (15)	C13—C12—H12	120.2
C5—C4—C3	105.95 (15)	C11—C12—H12	120.2
C5—C4—C15	123.41 (16)	C14—C13—C12	120.33 (15)
C3—C4—C15	130.63 (15)	C14—C13—H13	119.8
C4—C5—O1	110.84 (14)	C12—C13—H13	119.8
C4—C5—H5	124.6	C13—C14—C9	120.75 (16)
O1—C5—H5	124.6	C13—C14—H14	119.6
O2—C6—C3	119.83 (15)	C9—C14—H14	119.6
O2—C6—C7	120.92 (15)	C4—C15—H15A	109.5
C3—C6—C7	119.25 (14)	C4—C15—H15B	109.5
C8—C7—C6	120.31 (15)	H15A—C15—H15B	109.5
C8—C7—H7	119.8	C4—C15—H15C	109.5
C6—C7—H7	119.8	H15A—C15—H15C	109.5
C7—C8—C9	127.15 (15)	H15B—C15—H15C	109.5
			
C5—O1—C2—C3	−0.31 (17)	C2—C3—C6—C7	−164.79 (15)
C5—O1—C2—C1	178.43 (14)	C4—C3—C6—C7	15.8 (3)
O1—C2—C3—C4	0.56 (18)	O2—C6—C7—C8	6.9 (2)
C1—C2—C3—C4	−177.90 (17)	C3—C6—C7—C8	−173.80 (15)
O1—C2—C3—C6	−179.00 (14)	C6—C7—C8—C9	179.30 (15)
C1—C2—C3—C6	2.5 (3)	C7—C8—C9—C10	172.52 (16)
C2—C3—C4—C5	−0.59 (18)	C7—C8—C9—C14	−8.9 (3)
C6—C3—C4—C5	178.92 (17)	C14—C9—C10—C11	0.1 (3)
C2—C3—C4—C15	−179.55 (17)	C8—C9—C10—C11	178.70 (16)
C6—C3—C4—C15	0.0 (3)	C9—C10—C11—C12	−0.1 (3)
C3—C4—C5—O1	0.42 (18)	C10—C11—C12—C13	0.2 (3)
C15—C4—C5—O1	179.47 (15)	C11—C12—C13—C14	−0.2 (3)
C2—O1—C5—C4	−0.08 (18)	C12—C13—C14—C9	0.2 (3)
C2—C3—C6—O2	14.5 (2)	C10—C9—C14—C13	−0.1 (3)
C4—C3—C6—O2	−164.92 (16)	C8—C9—C14—C13	−178.66 (16)

### Hydrogen-bond geometry (Å, º)

*Cg*1 and *Cg*2 are the centroids of the furan (O1/C2–C5) and phenyl (C9–C14) rings, respectively.

**Table:**

*D*—H···*A*	*D*—H	H···*A*	*D*···*A*	*D*—H···*A*
C8—H8···O2	0.95	2.44	2.792 (2)	102
C10—H10···O2^i^	0.95	2.52	3.413 (2)	157
C12—H12···*Cg*1^ii^	0.95	2.91	3.7339 (19)	146
C15—H15*B*···*Cg*2^iii^	0.98	2.85	3.6366 (19)	137

Symmetry codes: (i) *x*+1/2, −*y*+3/2, −*z*+1; (ii) −*x*+3/2, −*y*+1, *z*−1/2; (iii) −*x*+1, *y*−1/2, −*z*+1/2.
